# Camphor, Applied Epidermally to the Back, Causes Snout- and Chest-Grooming in Rats: A Response Mediated by Cutaneous TRP Channels

**DOI:** 10.3390/ph12010024

**Published:** 2019-02-02

**Authors:** Débora T. Ishikawa, Robson Cristiano Lillo Vizin, Cristiane Oliveira de Souza, Daniel Carneiro Carrettiero, Andrej A. Romanovsky, Maria Camila Almeida

**Affiliations:** 1 Graduate Program in Neuroscience and Cognition, Federal University of ABC, São Bernardo do Campo 09606-070, SP, Brazil; idebor@gmail.com (D.T.I.); robson.vizin@gmail.com (R.C.L.V.); cristiane.souza@ufabc.edu.br (C.O.d.S.); daniel.carrettiero@ufabc.edu.br (D.C.C.); 2 Thermoregulation and Systemic Inflammation Laboratory (FeverLab), Trauma Research, St. Joseph’s Hospital and Medical Center, Phoenix, AZ 85013, USA; andrej.romanovsky@dignityhealth.org; 3 Center for Natural and Human Sciences, Federal University of ABC, São Bernardo do Campo 09606-070, SP, Brazil

**Keywords:** AMG8432, anxiety, behavioral thermoregulation, body temperature, camphor, heat defenses, heat exposure, ruthenium red, thermoregulatory behaviors, TRPV3

## Abstract

Thermoregulatory grooming, a behavioral defense against heat, is known to be driven by skin-temperature signals. Because at least some thermal cutaneous signals that drive heat defenses are likely to be generated by transient receptor potential (TRP) channels, we hypothesized that warmth-sensitive TRPs drive thermoregulatory grooming. Adult male Wistar rats were used. We showed that camphor, a nonselective agonist of several TRP channels, including vanilloid (V) 3, when applied epidermally to the back (500 mg/kg), caused a pronounced self-grooming response, including paw-licking and snout- and chest-“washing”. By the percentage of time spent grooming, the response was similar to the thermoregulatory grooming observed during exposure to ambient warmth (32 °C). Ruthenium red (a non-selective antagonist of TRP channels, including TRPV3), when administered intravenously at a dose of 0.1 mg/kg, attenuated the self-grooming behavior induced by either ambient warmth or epidermal camphor. Furthermore, the intravenous administration of AMG8432 (40 mg/kg), a relatively selective TRPV3 antagonist, also attenuated the self-grooming response to epidermal camphor. We conclude that camphor causes the self-grooming behavior by acting on TRP channels in the skin. We propose that cutaneous warmth signals mediated by TRP channels, possibly including TRPV3, drive thermoregulatory self-grooming in rats.

## 1. Introduction

Body temperature (T_b_) regulation is an important homeostatic function of ectothermic and endothermic animals alike [1]. It uses two major types of effector mechanisms: heat defenses and cold defenses. Both heat and cold defenses are represented by autonomic (or physiological; especially in endotherms) and behavioral (in all animals) effectors [2]. Thermoregulatory behaviors vary widely, and many of these are species-specific. Examples of thermoregulatory behaviors include nest-building (or house-building), postural adjustments, selection of a thermal environment, and self-grooming [2,3,4,5]. The latter is a robust heat-defense mechanism used primarily by rodents to spread saliva over the body surface for facilitating evaporative heat loss from the skin. It involves stereotyped behavioral acts, such as paw-licking, snout-washing, and body-rubbing, coupled with an autonomic response, salivation [3,6,7,8]. It has been shown that thermoregulatory grooming is driven by skin-temperature (T_sk_) signals [9,10,11]. Since at least some thermal cutaneous signals that drive thermoeffectors are likely to be generated by the so-called transient receptor potential (TRP) channels [12], we asked if TRP channels also drive thermoregulatory grooming. A priori, several warmth-sensitive channels can be involved, including those activated at temperatures that are characteristic of the vasodilated skin (>30 but <36 °C for rats) in a thermoneutral environment, such as the TRP channel melastatin 2 and the TRP channel vanilloid (V) 3. We paid special attention to the latter because we were able to obtain a novel experimental tool to probe its function: the TRPV3 antagonist AMG8432. The TRPV3 channel is prominently expressed in the skin (keratinocytes) and also reported in other epithelial cells across species, while its expression in the sensory neurons is low, at least in rodents [13]. TRPV3 opens in vitro at temperatures >33 °C [14,15]. Importantly, TRPV3 has been proposed to drive thermoregulatory behaviors in rodents, even though its role in such behaviors remains unclear (for a recent review, see [16]).

In this study, we investigated the involvement of warmth-sensitive TRP channels, including TRPV3, in the self-grooming (saliva-spreading) behavior induced by thermal and pharmacological stimulation in rats. Studies like this shed light on the organization of thermoregulatory behaviors and pave the way to what we have termed “thermopharmacology”—the approach of modulating thermoeffector activities and behaviors by drugs that mimic thermal stimulation [17]. 

## 2. Results

### 2.1. Exposure to Warmth Causes Thermoregulatory Self-Grooming

**Figure 1:**
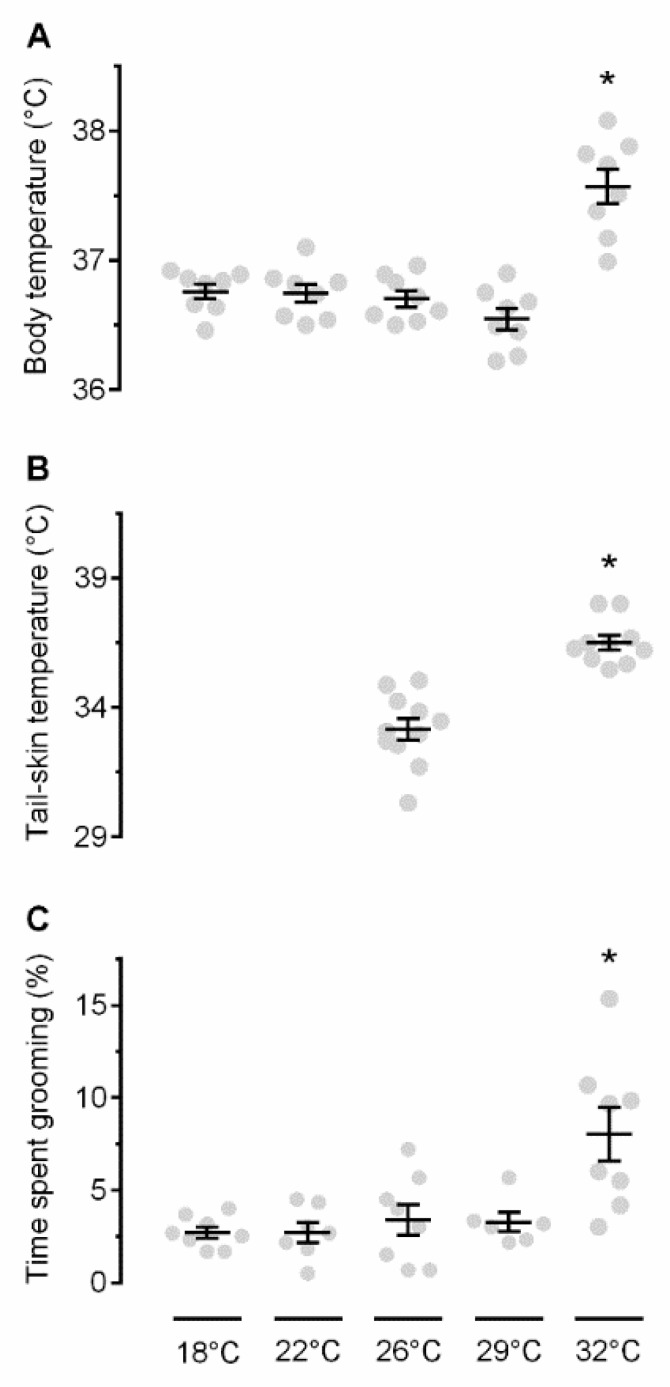
Effects of different T_a_s on T_b_ (**A**), T_sk_ (**B**), and grooming (**C**). An asterisk (*) indicates a significant difference (*p* < 0.05), as compared to the neutral T_a_ (26 °C).

First, we studied the self-grooming response to ambient warmth. Exposure to an ambient temperature (T_a_) in the range of 18–29 °C resulted in no T_b_ differences between the groups (*p* > 0.05; Figure 1A), whereas, at 32 °C, rats became hyperthermic (as compared to any other T_a_ used: 18 °C *p* < 0.001; 22 °C *p* < 0.001, 26 °C *p* < 0.001, or 29 °C *p* < 0.001). At the same T_a_, rats exhibited an increased T_sk_ (Figure 1B), signifying tail-skin vasodilation (*p* < 0.001 vs. 26 °C). Some basic level of self-grooming (2.7–3.4% of time), presumably hygienic in nature, was observed at 18–29 °C, with no statistical differences between the groups studied at different T_a_s (*p* > 0.05; Figure 1C). However, the self-grooming behavior became prominent (~8%) at 32 °C (*p* < 0.001 vs. 18 °C, *p* = 0.001 vs. 22 °C, *p* = 0.004 vs. 26 °C, *p* = 0.006 vs. 29 °C). Presumably, this was a warmth-defense response, because exposure to the same T_a_ resulted in a T_b_ increase and tail-skin vasodilation.

### 2.2. Camphor Induces Self-Grooming

**Figure 2:**
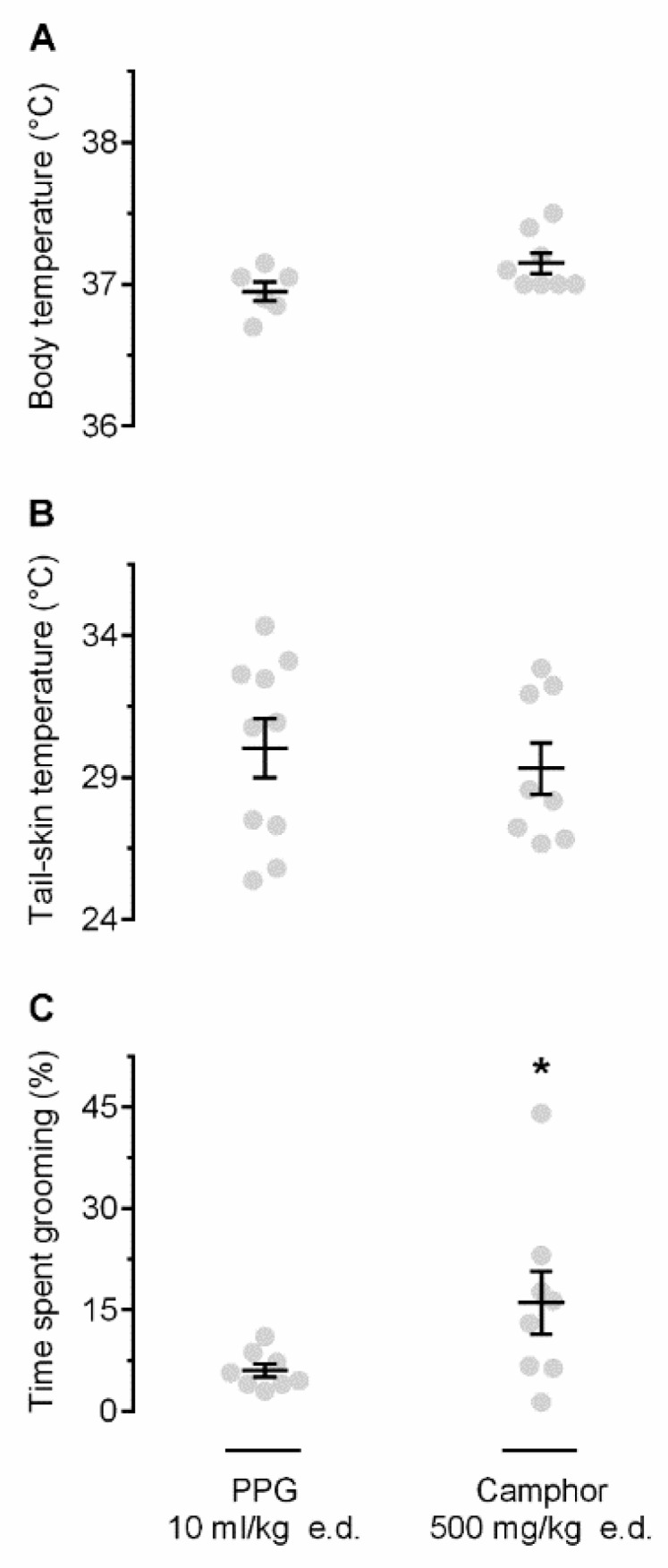
Effect of camphor on T_b_ (**A**), T_sk_ (**B**), and grooming (**C**). Camphor, a nonselective transient receptor potential (TRP) agonist, when applied epidermally (e.d.; dose indicated), did not alter T_b_ or T_sk_, but increased the time spent self-grooming, as compared to vehicle (polypropylene glycol, PPG). The experiments were conducted at neutral T_a_ of 26 °C. An asterisk (*) indicates a significant difference (*p* < 0.05), as compared to PPG-treated rats.

Next, we studied whether self-grooming can be induced by stimulating TRP channels. In these experiments, we used camphor, a nonselective agonist of several TRP channels, mostly warmth-sensitive, viz., TRPV1 [18] and TRPV3 [19]. We hypothesized that camphor would activate cutaneous warmth-sensitive TRP channels, thus mimicking the action of heat on the skin, and that these pharmacologically initiated cutaneous signals would cause self-grooming. To this end, camphor was applied to the skin of the back at 26 °C, i.e., at the T_a_ that caused no thermoregulatory grooming in the experiment described above (Figure 1C). At this T_a_, camphor (as compared to polypropylene glycol, PPG) did not cause any T_b_ changes (Figure 2A) or T_sk_ (Figure 2B), but it did trigger a robust self-grooming response (Figure 2C). The time spend self-grooming for camphor- vs. PPG-treated rats was 16.0 ± 4.7% vs. 6.0 ± 1.0% (*p* = 0.017). Importantly, during camphor-induced self-grooming, rats “washed” their fir on the snout, neck, and chest, i.e., the areas that had no exposure to camphor.

Self-grooming occurs not only as a thermoregulatory behavior, but also as a sign of stress and anxiety [20,21,22]. To test whether the self-grooming response to camphor was related to anxiety, we applied either camphor or PPG to the skin of the back and placed the rat in an elevated plus-maze apparatus. We found that, in this test, all behavioral responses to camphor were similar to those to PPG (Table 1). No significant difference was observed in the number of entries into the open or closed arms, the time spent inside the open or closed arms, or the number of events of rearing, head-dipping, or risk assessment between the two treatments. The number of fecal boli or urine spots did not differ between the treatments either. These data indicate that the self-grooming response to camphor (Figure 2C) was not due to anxiety.

Another possibility that had to be addressed was that camphor, a compound with a strong odor, caused the self-grooming behavior not by acting on thermoreceptors in the skin (topically), but by irritating the nasal mucosa (distantly). To test this possibility, we studied the self-grooming response to camphor (or PPG), when the substance was applied not to the skin, but to a cotton ball. The ball was placed in the cage and separated from the rat by mesh, thus, remaining out of the animal’s reach. Under these conditions, only the low, hygienic levels of self-grooming were observed, and no difference occurred between rats exposed to camphor or PPG (9.4 ± 1.6 vs. 9.7 ± 1.9% of time spent grooming; *p* > 0.05; data not shown). These data suggest that the self-grooming response to camphor was not induced by the odor, but rather was due to a topical, cutaneous action of this compound. We conclude that the nonselective TRP agonist camphor activates the thermoregulatory-like self-grooming behavior by acting on the skin.

### 2.3. Thermoregulatory Self-Grooming Is Mediated by TRP Channels

**Figure 3:**
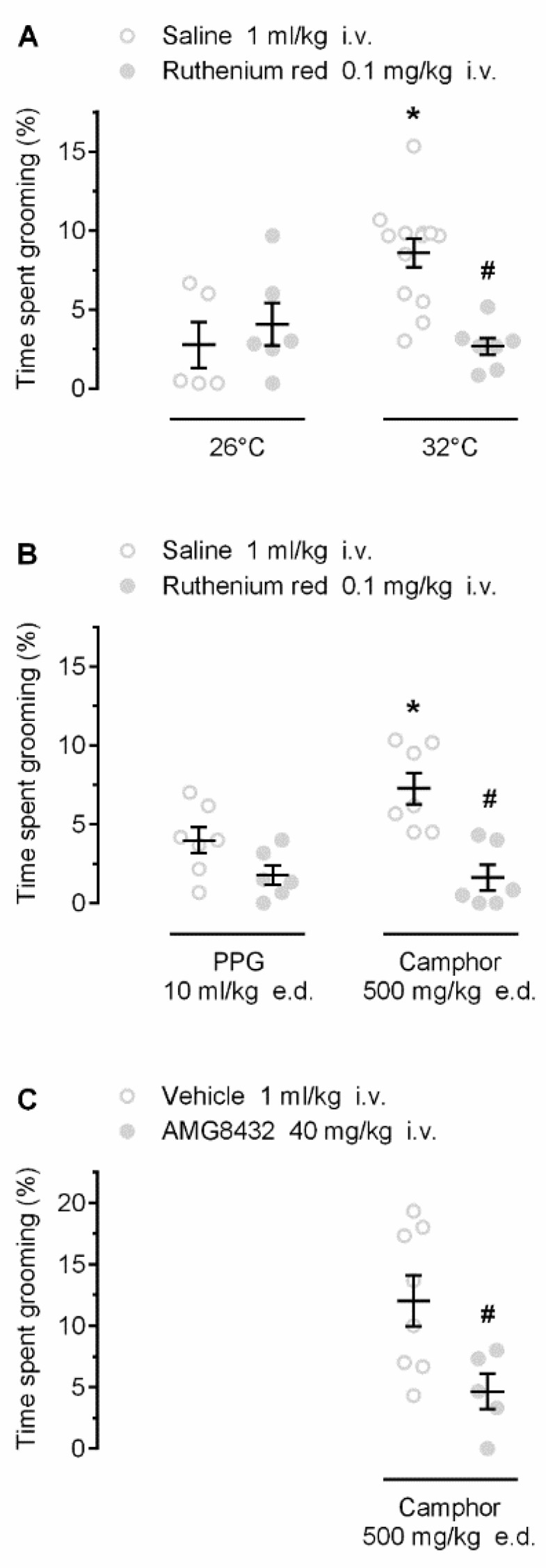
Effects of pharmacological blockade of TRP channels on warmth- (**A**) and camphor- (**B**,**C**) induced grooming. Warmth- and camphor-induced self-grooming responses were blocked by pretreatment with the nonselective TRP antagonist ruthenium red (dose indicated). Camphor-induced self-grooming was also blocked by AMG8432, a relatively nonselective TRPV3 antagonist. Both ruthenium red and AMG8432 were injected 30 min prior the exposure to 26 °C or 32 °C, or the camphor or PPG administration. An asterisk (*) indicates a significant difference (*p* < 0.05) between grooming responses at 32 vs. 26 °C (**A**), or in saline-pretreated camphor-treated vs. saline-pretreated PPG-treated rats (**B**). A pound sign (#) indicates a significant difference (*p* < 0.05) between grooming responses: in ruthenium red- vs. saline-pretreated rats at 32 °C (**A**); ruthenium red- vs. saline-pretreated camphor-treated rats (**B**); or in AMG8432-pretreated camphor-treated vs. vehicle-pretreated camphor-treated rats (**C**).

We then investigated whether the self-grooming responses to warmth and camphor are mediated by TRP channels. First, we studied whether ruthenium red, a nonselective TRP antagonist with marked potency against several warmth-sensitive rat channels (including TRPV1, TRPV2, TRPV3, and TRPV4 [23,24]), would attenuate the warmth- and camphor-induced grooming. As expected, warmth ambient (Figure 3A) and camphor (Figure 3B) induced self-grooming in saline-pretreated rats (*p* < 0.001 and *p* = 0.009, respectively). Pretreatment with ruthenium red attenuated the grooming responses to both stimuli (*p* < 0.001; Figure 3), but not the low-level hygienic grooming observed at 26 °C (*p* = 0.476) or in response to PPG (*p* = 0.079). These results suggest that the grooming behavior occurring in response to ambient warmth or camphor is mediated by TRP channels. 

One potential development of this study, as pointed out by the reviewers of this manuscript, would be to assess the involvement of TRPV4 channels. However, in our earlier study [25], the selective TRPV4 antagonist RN-1734 (2,4-dichloro-N-isopropyl-N-(2-isopropylaminoethyl) benzenesulfonamide) [26] did not affect the self-grooming behavior in rats. In that study, RN-1734 (Tocris Bioscience, Minneapolis, MN, USA) was administered intravenously (i.v.) at a dose of 500 μg/kg 30 min before the self-grooming response to ambient warming (T_a_ of 32 °C) was evaluated. Furthermore, TRPV4 is activated by somewhat lower temperatures (<30 °C) [27] than TRPV3. It is also noteworthy that camphor, which was used to induce grooming in the present study, has not been reported to activate TRPV4. Hence, we did not study the involvement of TRPV4 in the present investigation.

Next, we used AMG8432, a novel, relatively potent TRPV3 antagonist with limited selectivity (see Materials and Methods), and tested whether it would attenuate camphor-induced self-grooming. Rats pretreated with AMG8432 showed a significant reduction (by ~7.4%) in camphor-induced self-grooming, as compared to vehicle (*p* = 0.026; Figure 3C). These results suggest that the self-grooming response to camphor is mediated by TRP channels, possibly including TRPV3.

## 3. Discussion

In the present study, we used camphor, a bicyclic monoterpene ketone typically isolated from the wood of the camphor laurel tree (*Cinnamomum camphora*). Camphor activates several rat TRP channels, most notably warmth-sensitive, viz., TRPV1 and TRPV3 [18,19,28]. This compound also sensitizes the TRPV3 channel [19,29,30] and modulates the sensation of warmth in humans [31]. We have shown that camphor, when applied to the back of a rat, causes marked grooming of other areas of the body (the snout and chest), a response that appears to be thermoregulatory. Interestingly, the intensity of the grooming response to camphor (measured as the percent of time spent grooming) was nearly identical to the intensity of the grooming response to ambient heating (compare Figure 1C and Figure 2C). The grooming response to camphor was not caused by the odor of the compound, as confirmed by the observation that rats did not respond with grooming to the camphor odor when no camphor was applied to the skin. Neither was the grooming response to camphor associated with anxiety, as evident from the fact that camphor did not trigger any anxiety-like behaviors in the elevated plus-maze test, as compared to vehicle. Therefore, we conclude that camphor drives self-grooming by acting on the skin, and that response is not related to anxiety.

We have further shown that the grooming response to camphor was attenuated by ruthenium red, a nonselective antagonist of several TRP channels, including the warmth-sensitive rat channels TRPV1, TRPV2, TRPV3, and TRPV4 [23,32]. In this regard, the camphor-induced grooming appeared to be similar to the thermoregulatory (ambient warmth-induced) grooming, as both behaviors were attenuated by ruthenium red. 

It is well-known that thermoregulatory grooming is driven by cutaneous warmth signals [9,10,11]. Combining this knowledge with the present data, it is tempting to conclude that both warmth- and camphor-induced grooming are mediated by cutaneous warmth-sensitive TRP channels.

Because camphor is well recognized for activating and sensitizing the TRPV3 channel [19,29,30], whereas ruthenium red is a nonselective and reasonably potent TRPV3 antagonist [14,15,33], our attention became focused on the TRPV3 channel. In heterologous systems in vitro, TRPV3 opens at temperatures >33 °C [14,15], i.e., within the upper range of physiological values of T_sk_, as observed during skin vasodilation in rats [34]. Furthermore, TRPV3 has been reported to mediate the cutaneous vasodilatation induced by local heating and to play a role in defending the deep T_b_ during heat exposure [35]. Other studies suggest that TRPV3 is involved in the avoidance of innocuous ambient heat in transgenic mice, as well as in thermal pain responses [19,36]. Yet, TRPV3 knockout mice show no obvious thermoregulatory phenotype [37] and, specifically, no alterations in the deep T_b_ [19,35]. Overall, there is no consensus as to whether TRPV3 is important in driving thermoregulatory responses, and which responses in particular [16]. In the present work, we showed that AMG8432 reduced the self-grooming response to camphor. Because the potency of this compound is by an order of magnitude higher against the TRPV3 channel than other warmth-sensitive TRP channels (TRPV1 and TRPV4), we speculate that TRPV3 may mediate the grooming response. To prove this hypothesis definitively, further research is needed, and experiments with additional, more selective, TRPV3 antagonists (when they become available) and genetic tools (TRPV3 knockout, siRNA, or lentiviral shRNA) can be instrumental. 

In summary, we show that camphor (a nonselective agonist of several warmth-sensitive TRP channels, including TRPV3), applied to the skin, causes a self-grooming response, which is similar to thermoregulatory grooming. We have further shown that ruthenium red (a nonselective antagonist of several TRP channels, including TRPV3) attenuates the grooming response to either ambient warmth or camphor, and that AMG8432 (a relatively nonselective TRPV3 antagonist) attenuates the camphor-induced self-grooming behavior. Together, these data suggest that, in rats, TRP channels, possibly including TRPV3, mediate those cutaneous warmth signals that drive thermoregulatory self-grooming.

## 4. Materials and Methods 

### 4.1. Animals 

Male Wistar rats (9–10 weeks old; 290–320 g) were obtained from the Institute of Biomedical Sciences, University of São Paulo (São Paulo, SP, Brazil). All rats were housed in standard cages kept in ventilated cabinets (Alesco, Monte Mor, SP, Brazil) under a 12:12 h light-dark cycle (lights on at 7 AM) and a controlled T_a_ of 22–24 °C. Standard laboratory rodent chow and tap water were available ad libitum (except during the experiments). All experiments were performed between 9 AM and 2 PM. All procedures were approved by the ethics committee of the Federal University of ABC (protocols 001/2010 and 003/2013).

### 4.2. Surgery

#### 4.2.1. General

For all surgical procedures, rats were anesthetized with an intraperitoneal administration of ketamine-xylazine cocktail (75 mg/kg and 10 mg/kg, respectively) and treated with an antibiotic (enrofloxacin, 15 mg/kg, intramuscularly) and a nonsteroidal anti-inflammatory drug (flunixin meglumine, 2.5 mg/kg, subcutaneously). Experiments were performed 5–7 days after surgery. At the end of the experiments, rats were euthanized with urethane 30% (10 mL/kg, intraperitoneally).

#### 4.2.2. Temperature-Measuring Device Implantation

Via midline laparotomy, each rat was implanted with a datalogger (Sub Cue, Calgary, AB, Canada) in the peritoneal cavity for recording abdominal temperature, a measure of deep Tb. The abdominal wall was sutured in layers. After the experiments, the rat was euthanized, the datalogger was removed, and the Tb data were uploaded to a computer for processing. 

#### 4.2.3. Jugular Catheterization

Each rat assigned to experiments involving i.v. injections was also implanted with a jugular catheter. A small incision was made on the ventral surface of the neck, to the right of and parallel to the trachea. The right jugular vein was exposed, freed from its surrounding connective tissue, and ligated. A silicone catheter (inner diameter: 0.5 mm; outer diameter: 0.9 mm), filled with heparinized (10 U/mL) saline, was passed into the superior vena cava through the jugular vein and secured in place with ligatures. The free end of the catheter was knotted, tunneled under the skin to the nape, and exteriorized. The wound was sutured. The catheter was flushed with heparinized saline the day after surgery and every other day thereafter, up to the day of the experiment. After catheterization, rats were housed individually. 

### 4.3. Drugs

Camphor, a nonselective TRP agonist [18,19], was dissolved in PPG to a concentration of 5%, wt/vol (working solution). Camphor (500 mg/kg) or PPG (10 mL/kg) was applied e.d., to the unshaved skin on the back of a rat, by spreading the solution in the tail-to-head direction. Importantly, the area of application cannot be reached by a rat, and, hence, a rat cannot further spread the applied substance over the body. Ruthenium red, a nonselective TRP blocker [23], was purchased from Tocris Bioscience. It was dissolved in saline to a concentration of 0.1 mg/mL (working solution) and administrated as a bolus (1 mL/kg i.v.). Ruthenium red (or saline) was administered at 30 min before the onset of heat exposure or the e.d. administration of camphor. The number of pharmacological tools to work with the TRPV3 channel is still very limited [13]. For this study, we were able to obtain AMG8432, a (relatively selective) TRPV3 antagonist (donated by Amgen, Thousand Oaks, CA, USA). AMG8432 exhibits the highest potency against human TRPV3 (IC50 for 2-aminoethoxydiphenyl borate is 1 μM) with some, substantially lower, activity against human TRPV1 (IC50 for capsaicin is >10 μM) and TRPV4 (IC50 for 4α-phorbol 12,13-dicaprinate is 12 μM). All IC50 values (in µM, rounded to the nearest whole number) were kindly provided by Amgen (Sonya G. Lehto and Bryan D. Moyer, personal communication). AMG8432 was dissolved in 30% DMSO, 35% PPG, and 35% ethanol to a concentration of 40 mg/mL (working solution). AMG8432 was slowly infused (40 mg/kg, 33.3 µL/kg/min i.v.) over a period of 30 min using a micro infusion pump (Harvard Apparatus, Holliston, MA, USA). AMG8432 (or its vehicle) was administered i.v. at 30 min before the e.d. administration of camphor. The paradigm chosen for drug administration, i.e., an antagonist (ruthenium red or AMG8432) i.v. followed by an agonist (camphor) e.d., was chosen to avoid direct interactions between the drugs (and their vehicles); this paradigm was successfully used in our work with other TRP channels (e.g., melastatin-8 [17]) in the past.

### 4.4 Experimental Setups 

#### 4.4.1. Experimental “Arena” for Behavioral Studies

An acrylic arena (30 × 30 × 30 cm, width × length × height, respectively), lined with wood bedding, was set inside a climatic chamber (Eletrolab; São Paulo, SP, Brazil), at a T_a_ of 18, 22, 26, 29, or 32 °C. The midpoint of the thermoneutral zone for rats in this setup is ~26 °C [38,39]. During an experiment, a rat was placed in the arena for behavioral observations. Prior to experiments, rats were extensively habituated to the arena.

#### 4.4.2. Elevated Plus-Maze Apparatus

Observations in an elevated plus maze are often used to assess anxiety-like behaviors in rats [40]. We used an elevated plus-maze apparatus made of white wood and consisted of a central square (10 × 10 cm) and four arms (10 × 50 cm each) radiated outward from the square. Two mutually opposite arms were “closed”, i.e., had 40-cm high walls. The other two mutually opposite arms were “open”, i.e., did not have walls. The maze was elevated to 55 cm off the floor. A rat was placed on the central platform of the apparatus facing one of the closed arms.

### 4.5. Parameters Measured

Deep T_b_ (abdominal) was recorded with implanted dataloggers every 5 min. Tail T_sk_, a measure of the tail vasomotor tone, was determined based on the images taken with an A300 infrared camera (FLIR, Portland, OR, USA). The values of T_sk_ were obtained at the border of the proximal and middle thirds of the tail. Large (several degrees C) increases in T_sk_ typically signify tail-skin vasodilation [2,34]. The self-grooming behavior in the arena and anxiety-like behaviors in the elevated plus-maze apparatus were analyzed based on 5-min-long video recordings. When rats were exposed to different T_a_s (18–32 °C), the recording started after 2 h of exposure. In experiments with camphor (or PPG) administration, the recording started at 5 min after the administration. The self-grooming response was measured as a percentage of time a rat spent engaging in this behavior. In the elevated plus maze, 5 min after the camphor (or PPG) treatment, rats were individually placed in the apparatus and the following indices of anxiety were studied: an increased time spent in and number of entries to the closed arms; a decreased time spent in and number of entries to the open arms; an increased number of events of “risk-assessment behavior” (when a rat stands on hind paws on the central platform, while stretching its body to “peek” into an open arm); head dipping (when a rat stretches its body beyond the edge of an open arm and lowers the head below the surface of the arm); and rearing, as well as increased numbers of fecal boli and urine spots.

### 4.6. Data Analysis

One-way ANOVA and two-way ANOVA were used, followed by the Holm–Sidak post-hoc test, as appropriate. Student’s *t*-test was used to compare single-measurement values between the two groups. All analyses were performed using Sigma Plot 13.0 (Systat Software). Differences were considered significant at *p* < 0.05. Data are reported as Mean ± SE.

## Figures and Tables

**Table 1:** Effects of camphor or its vehicle (PPG) on behaviors in elevated plus maze.

Behavioral Index	PPG (n = 7)	Camphor (n = 7)	*p* Value
Entries to open arms	1 ± 0	1 ± 0	0.82
Entries to closed arms	3 ± 1	5 ± 1	0.18
Time in open arms (s)	19 ± 6	35 ± 13	0.29
Time in closed arms (s)	244 ± 10	234 ± 15	0.58
Rearing events	10 ± 1	11 ± 2	0.79
Head-dipping events	2 ± 1	3 ± 1	0.21
Risk-assessment events	6 ± 1	6 ± 1	0.82
Fecal boli	3 ± 1	4 ± 1	0.29
Urine spots	2 ± 1	2 ± 1	0.91

## Abbreviations

e.d.	epidermal(ly)
i.v.	intravenous(ly)
T_a_	ambient temperature
T_b_	body temperature
T_sk_	skin temperature
TRP	transient receptor potential (channel)
PPG	polypropylene glycol
V	vanilloid (as in TRPV3)
